# Increased interpretation of deep learning models using hierarchical cluster-based modelling

**DOI:** 10.1371/journal.pone.0295251

**Published:** 2023-12-07

**Authors:** Elise Lunde Gjelsvik, Kristin Tøndel

**Affiliations:** Faculty of Science and Technology, Norwegian University of Life Sciences, Aas, Norway; NUST: National University of Sciences and Technology, PAKISTAN

## Abstract

Linear prediction models based on data with large inhomogeneity or abrupt non-linearities often perform poorly because relationships between groups in the data dominate the model. Given that the data is locally linear, this can be overcome by splitting the data into smaller clusters and creating a local model within each cluster. In this study, the previously published Hierarchical Cluster-based Partial Least Squares Regression (HC-PLSR) procedure was extended to deep learning, in order to increase the interpretability of the deep learning models through local modelling. Hierarchical Cluster-based Convolutional Neural Networks (HC-CNNs), Hierarchical Cluster-based Recurrent Neural Networks (HC-RNNs) and Hierarchical Cluster-based Support Vector Regression models (HC-SVRs) were implemented and tested on spectroscopic data consisting of Fourier Transform Infrared (FT-IR) measurements of raw material dry films, for prediction of average molecular weight during hydrolysis and a simulated data set constructed to contain three clusters of observations with different non-linear relationships between the independent variables and the response. HC-CNN, HC-RNN and HC-SVR outperformed HC-PLSR for the simulated data set, showing the disadvantage of PLSR for highly non-linear data, but for the FT-IR data set there was little to gain in prediction ability from using more complex models than HC-PLSR. Local modelling can ease the interpretation of deep learning models through highlighting differences in feature importance between different regions of the input or output space. Our results showed clear differences between the feature importance for the various local models, which demonstrate the advantages of a local modelling approach with regards to interpretation of deep learning models.

## Introduction

For applications where interpretability is important, latent variable based models such as Partial Least Squares Regression (PLSR) [1] have clear advantages over neural networks. However, for complex data sets with large inhomogeneity, linear prediction models like PLSR often perform poorly. In these cases, the relationships between groups in the data dominate the model and the prediction approaches the average of the closest group [2]. Poor prediction can also occur when there are several different competing interrelationships overshadowing the prediction problem of interest. An important step in achieving a good prediction model is therefore to identify and model these underlying structures as real world data often have unknown structures and hidden relationships which can be difficult to model. To overcome this problem, the data can be split into smaller clusters of more homogeneous data. If the split is successful, the estimation of the relationships among both variables and observations will achieve an improved prediction. The split can be based on any criterion, i.e. be manually determined or assigned by a clustering algorithm. Clustering can find the hidden structures without having any prior knowledge about the data. This approach yields a set of models, one for each cluster, where each of them are fitted to the data within that cluster, decreasing the shortcomings of a model based on all the data.

Local linear approaches for modelling large and complex data sets have been shown to work well, and PLSR has been used in several studies where the data has been divided based on prior knowledge [2–8]. However, the data does not always have known groups to base the separation on, creating a need for other approaches. For instance, Tøndel *et al*. [9, 10] created a hierarchical PLSR model (HC-PLSR), where the observations were assigned into clusters based on Fuzzy C-Means (FCM) clustering which assumes no prior knowledge about the data structure. Fuzzy clustering methods have also been developed within a framework that determines the optimal number of clusters [11, 12].

The advantage of PLSR is its efficient ways of finding latent variables in the data fast and reliably. Nevertheless, a potential pitfall when it comes to PLSR, is that this is a linear model, while the data could have non-linear interrelationships where PLSR will struggle to perform well. The input space the data lies in can exist in different planes, be high-dimensional, low-dimensional, linear, non-linear etc. Even though simple non-linearities can be accounted for in PLSR by e.g. including polynomial terms in the regressor matrix, and more abrupt non-linearities can be handled by local modelling, some types of non-linearities can not be modelled using PLSR. In such cases, there is a need for a method able to handle more complex structures, as even with HC-PLSR, it is a requirement that the data is at least locally linear.

Neural networks are powerful when it comes to modelling large, non-linear data sets. However, the more complex the data becomes, the deeper the network needs to be to achieve adequate prediction. A deep network contains numerous parameters and uses large amounts of computational power to converge. For local modelling, smaller networks can be implemented without loosing predictability, rather increasing predictive power, and provided that the number of local models is kept relatively low, the risk of overfitting decreases. Interpretability of simpler models is higher, and coupled with clustering, an opportunity to identify regional differences in feature importance arises.

In this study, the framework for HC-PLSR was extended to deep learning based models in an attempt to improve the prediction and interpretability of complex non-linear data. Hierarchical Convolutional Neural Networks (CNNs), Recurrent Neural Networks (RNNs) and Support Vector Regression (SVR) models were created and compared to the HC-PLSR models. The HC modelling uses PLSR as the first step since this is a linear, easily intrepretable method. The clustering part of the hierarchical modelling is done on the latent variables from a global PLSR model. This provides an opportunity to interpret the data in a lower-dimensional subspace spanned by the latent variables, before local models are made within each of the clusters. However, with PLSR, this does not yield particularly high prediction abilities when the data within the clusters is non-linear, which was the motivation for expanding the HC-PLSR framework to HC deep learning. Visualisation and interpretation of these latent variables from the global models provide overview of covariation patterns in the data, which can pinpoint important differences in the properties of the clusters, and assist in identifying the underlying reasons for differences in feature importance between the clusters.

## Materials and methods

### Fuzzy C-Means clustering (FCM)

Cluster analysis consists of assigning data into groups in a way that the data points in the same group are as similar to each other and as dissimilar to data points in other groups as possible. These clusters are defined based on a similarity measure, for instance, distance, connectivity or intensity. The original HC-PLSR algorithm utilises FCM to form clusters. In Fuzzy clustering, each data point can belong to more than one cluster, where cluster membership probabilities define to which degree a sample belongs to the different clusters. The closer to the centroid the sample is, the higher the membership probability becomes. The FCM algorithm [13, 14] finds a given number of clusters where probabilities for being in the clusters are assigned randomly to each data point. This is repeated until the algorithm has converged, i.e. until the changes in the probabilities no longer exceed the set sensitivity threshold. The centroid is then computed for each cluster and finally, the membership probabilities for each sample for being in each of the clusters are computed.

### Alternative clustering techniques

Spectral clustering (SPC) [15] uses the spectrum (eigenvalues) of the similarity matrix of the data to reduce dimensionality, so that the clustering can be done in fewer dimensions. The similarity matrix consists of an assessment of the relative similarity for each pair of points in the data set. SPC is useful when the structure of the clusters is non-convex, when the center and spread of the cluster give a poor description of the properties of the cluster.

In hierarchical agglomerative clustering (HAC) [16], nested clusters are built by successive merging. This hierarchy of clusters can be presented as a dendrogram, where clusters are combined stepwise until all samples of the data set are incorporated into one cluster, the root. The distance between two subsets of the data is called the linkage distance and represents the distance between samples in the clusters, and thereby also the cluster regions.

### Partial Least Squares Regression (PLSR)

PLSR and its algorithm have previously been described rigorously in the literature [1, 17–19]. In short, PLSR decomposes large data sets into a subspace of latent variables (scores and loadings) representing the main features of covariance between ***X*** (regressors) and ***Y*** (responses), where both ***X*** and ***Y*** can be multivariate. PLSR uses inter-correlations between the response variables to stabilise the model, and does not require that the variables are linearly independent. The latent variables, the PLS components (PCs), represent the most relevant subspaces of the regressors. This is beneficial, as it makes PLSR able to handle a wide range of data, including chemometric data, where the number of features often exceeds the number of samples.

### Convolutional Neural Networks (CNNs)

CNNs are deep neural networks which use convolutions to extract information in one or more hidden layers [20, 21]. CNNs are regularized versions of fully connected networks and consist of an input layer, hidden layers (mainly convolutional layers, pooling and fully connected layers) and an output layer. In the convolutional layers, the data is organised in a feature map where the weights are connected to the previous layer. These weights are used to filter for patterns in the data. The pooling layer semantically merges similar features, reducing the dimension of the representation [21]. Commonly used in pattern recognition, CNNs are good feature extractors as they learn the most important features by themselves. In contrast to PLSR, CNNs can handle highly non-linear data.

### Recurrent Neural Networks (RNNs)

RNNs are neural networks often used for sequential or time-series data, feeding the output from one layer as input to the next layer [22, 23]. Like CNNs, the RNNs learn from the training input, but the RNNs use internal states (memory) to impact inputs and outputs with previous information. Therefore, RNNs have strong capabilities of capturing contextual data from a sequence. In an RNN, the input sequence is processed one element at a time with the memory in the hidden units retaining information on all the elements in the sequence [21]. In the case of chemical spectra, the peaks are often related to adjacent peaks or can appear in certain patterns, which is why pattern recognition methods such as CNNs and RNNs often achieve good prediction models for this type of data.

### Support Vector Regression (SVR)

In SVR, a hyperplane or a set of hyperplanes are constructed in a high-dimensional space to separate the observations in the training set [24–26]. The aim is to find the hyperplane that has the largest distance (margin) to the nearest data point. The margin is defined as the distance between the separating hyperplane, the decision boundary, and the training samples that are closest to this hyperplane. The hyperplane is used to predict the continuous output and the regression solution is the hyperplane that has the maximum number of data points. Decision boundaries with large margins tend to have a lower generalisation error, while decision boundaries with small margins are more prone to overfitting. This makes SVRs proficient at handling non-linearities in data, and as the hyperplanes are constructed in a high-dimensional space, SVR can handle data which cannot be separated in the first two or three dimensions.

### Hierarchical cluster-based modelling

**Fig 1:**
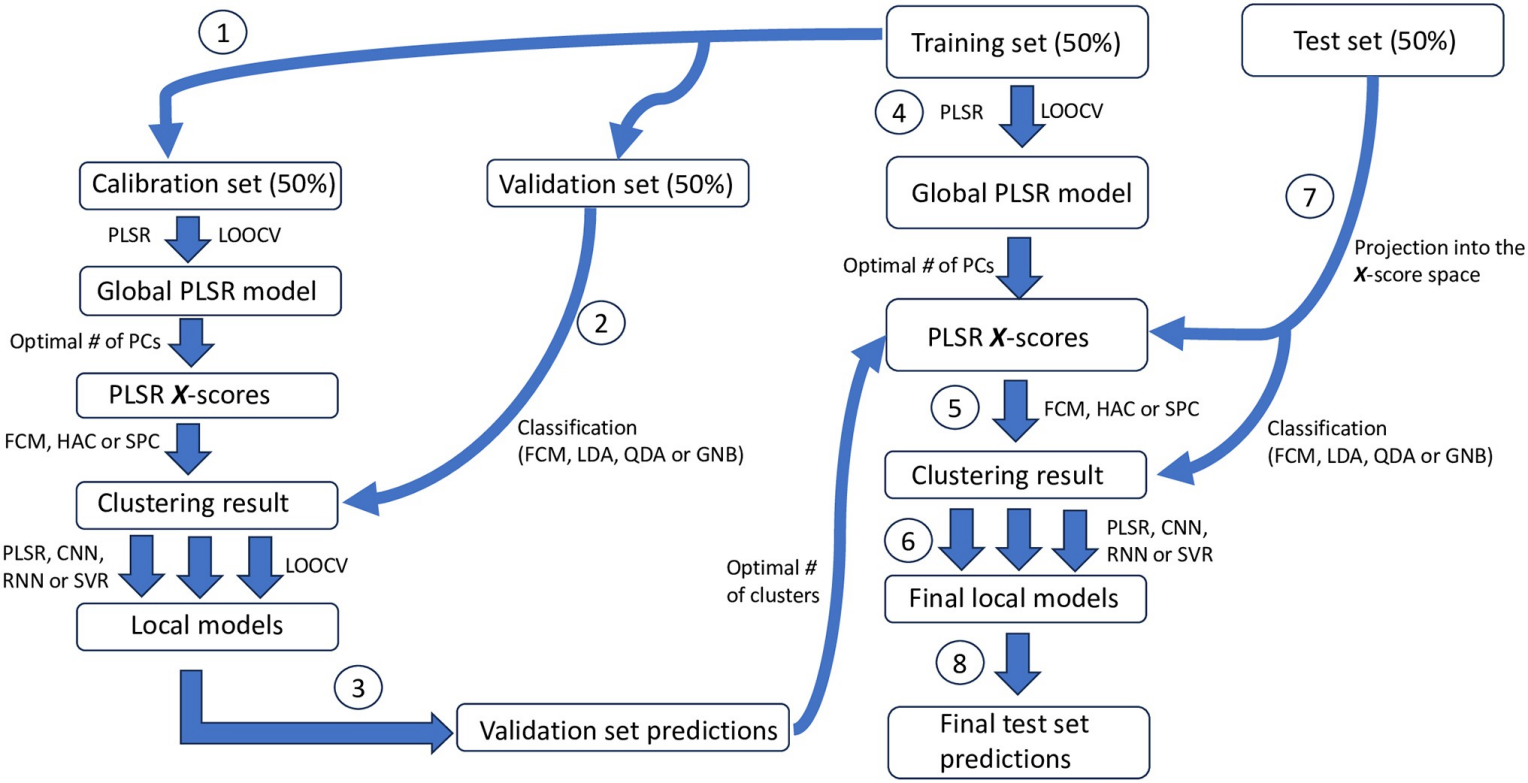
Illustration of the hierarchical cluster-based modelling procedure. The different parts of the algorithm are numbered according to the order at which they are carried out.

Implementation of the local modelling was based on the HC-PLSR procedure developed by Tøndel *et. al* [9], and is illustrated in Fig 1. Prior to the data modelling, the data was split into a training set (50%) and a test set (50%). The test set was kept totally separate, and only used for a final testing of the prediction abilities of the developed models.

All statistical methods were implemented using Python 3.8 and its machine learning packages. FCM was implemented using the fuzzy-c-means package [27]. The developed code can be obtained from the authors upon request. All calculations were done on the Orion High Performance Computing Center (OHPCC) at the Norwegian University of Life Sciences (NMBU).

#### Calibration of the global PLSR model

A PLSR model was first built using all observations in the training set, and the optimal number of PCs was determined using cross validation (Leave-One-Out cross validation (LOOCV) for the real world data set and 10-fold cross validation for the simulated data set) to reduce the risk of overfitting. The number of PCs to include was chosen based on the minimum cross-validated mean squared error (MSE) over the cross-validated models.

#### Clustering and calibration of the local models

The ***X***-scores for the samples from the global PLSR model were then clustered by FCM using Euclidean distance. Euclidean distance is also applied in the original HC-PLSR study [9]. The purpose of the local modelling is to capture the differences between the ***X***-***Y*** relationships in different parts of the data, and the PLS components explaining the largest parts of the covariance between ***X*** and ***Y*** are therefore the most relevant for the clustering.

In addition to FCM, several other clustering methods were fitted to the data to evaluate the clustering proficiency. HAC and SPC showed some interesting clusters that differed from FCM, and these methods were therefore implemented in the algorithm. The results from the local modelling using HCA and SPC for clustering were compared to the results obtained using FCM. The remaining clustering methods did not yield meaningful groupings of the data and were therefore rejected. All cluster distributions are presented in the S1 File.

For each of the determined clusters, local PLSR, CNN, RNN and SVR models were calibrated individually using LOOCV or 10-fold CV to find the optimal local model parameters (PCs for PLSR, training length and epochs for CNN and RNN, and hyperparameters for SVR) based on the minimum MSE.

#### Calibration of classification models

Classification models predicting the cluster memberships from the ***X***-scores were calibrated using LOOCV or 10-fold CV on the training set. Four different classification methods were implemented in our algorithm: FCM, Linear Discriminant Analysis (LDA) [28], Quadratic Discriminant Analysis (QDA) [29] and Naive Bayes classification (NB) [29]. In the cross validation, the kept-out samples were projected into the ***X***-score space of a PLSR model made using the remaining samples, their ***X***-scores were predicted, and used to find the cluster membership from a classification model made using the kept-in samples.

#### Prediction of the response for new observations

New (test set) observations, were projected into the global PLSR model and their ***X***-scores were calculated. The resulting ***X***-scores were then classified into the appropriate clusters based on FCM, LDA, QDA or NB.

Prediction of the response for the new observations was done using the local model for the assigned cluster. The resulting predictions were compared to those obtained using the global PLSR, CNN, RNN and SVR models to evaluate the improvements achieved using the local modelling.

In the original HC-PLSR algorithm, the opportunity to do a weighted prediction is included, where the final prediction is a weighted sum of the local model predictions, using the membership probabilities from FCM as weights. This option was excluded in the implementation presented here, since experiences have shown that using the closest local model for prediction has given better results.

#### Determination of the optimal number of clusters

In a separate procedure to determine the optimal number of clusters (numbered 1–3 in Fig 1), the training set was divided into a calibration set (50%) and a validation set (50%). The calibration set was used to calibrate the hierarchical models using LOOCV (with the same procedure as described above), and subsequent validation was done using the validation set. This was done to validate the classification and reduce the risk of overfitting from the LOOCV parameter optimisation. For clusters containing less than 10 samples, the samples were reassigned based on their membership probabilities, until they were placed in a cluster with more than 10 samples. The number of clusters determined to be optimal was then used to train the hierarchical models on the entire training set before final prediction on the test set that had been kept totally separate from the data used to train the models.

### Visualising important features

To interpret the resulting models, i.e. which ***X***-***Y*** relationships dominate the models, we used the following procedures to visualise the most important features in our real-world data set: For PLSR, the ***X***-loadings were used directly as feature importance measures, since they give an overview of which features that account for the explained variation in the response.

To evaluate which features the CNN and RNN estimated to be important during building of the hierarchical models, we visualised the gradients for each of the local models. This visualisation was based on the variational gradient method (VarGrad) [30, 31], which previously has shown good results compared to similar visualisation methods [32]. Jenul *et al.* showed that this method works well for determining the importance of blocks in a multiblock data set [33], and this procedure was therefore adapted to determine the importance of features. In VarGrad, a small random noise (using the Numpy Random Generator) is added to the input layer and then the gradient function for each feature is estimated. The resulting variation in the gradient indicates which of the features the output from the network is most dependent on. These features are deemed important, and their effects on the prediction can be evaluated.

For SVR, we chose to use permutation feature importance due to its widespread use. Permutation feature importance is a model inspection technique that identifies important features based on changes in the prediction ability when a feature is randomly shuffled (permuted) [34]. If the prediction accuracy of the model decreases significantly when a feature is randomly shuffled, this indicates that the feature is important for the model’s ability to predict the response. Similarly, if the prediction accuracy is unaffected, the feature is not important for the prediction. Alternatively, pseudo-sample projection could have been used to obtain the feature importance [35–38] from the SVR modelling.

In order to evaluate to what extent the various regions in the input space represent different ***X***-***Y*** relationships, the feature importance maps for each of the local models were compared to each other and to that resulting from the global model.

### Simulation study

To evaluate the proficiency of the developed local modelling methodology, a simulation study was performed. A data set consisting of 1500 samples with 20 features was created using the function make_blobs in Python and the response data was generated using the Python-functions make_friedman 1, 2 and 3 [39] (Scikit-learn [40]). These functions create ***X***-data and a response, with non-linear relationships between ***X*** and ***Y***. The data set was created so that it contained three defined clusters with ***Y***-data generated using make_friedman1, make_friedman2 and make_friedman3, respectively, to evaluate whether the hierarchical modelling was able to identify these clusters and use them to give a better prediction ability than for a global model. A plot of the simulated data set can be found in the S1 File. The data was randomly divided into training and test sets with half of the data set (50%) in each. Determination of the parameters for the global PLSR model and all hierarchical models was carried out by 10-fold cross-validation using the training set, while the final prediction was done using test set validation.

Visualisation of feature importance was not performed for the simulated data set as all the variables were designed to be random. For instance, permuting one variable to evaluate the effect on the prediction ability would only generate a new data set of random variables, and the feature importance would not be possible to interpret.

### Real world data set

The real world data set used to further compare the methods consists of Fourier-transform infrared spectroscopy (FT-IR) measurements of raw material dry films from chicken, turkey, salmon and mackerel hydrolysed by six different enzymes, a data set which was published by Kristoffersen *et al.* [4]. The pre-processing was performed using Savitzky-Golay [41] 2^nd^ derivative smoothing (with window width 11 pt and 3^rd^ order polynomial smoothing) followed by extended multiplicative signal correction (EMSC) [42] with 2^nd^ order polynomial correction, with the mean spectrum as reference. Lastly, the spectra were cut to contain the region between 1800 cm^-1^ and 700 cm^-1^ based on prior knowledge about the relevance of different parts of the spectra. This pre-processing was used in the original paper [4] and we selected the same processing to maintain comparability.

All samples were measured in replicates, and the average spectrum was calculated over the replicates for each sample, resulting in 332 unique samples. Different methods for processing the raw material before the enzymatic hydrolysis, four for chicken material, two for turkey material and one each for salmon and mackerel, were tested in the original study. The resulting data set included information about 28 different subgroups consisting of the six enzymes used for hydrolysis combined with the four raw materials and their various treatments. The response to be predicted was the average molecular weight (M_w_) during the enzymatic hydrolysis of the raw materials.

**Fig 2:**
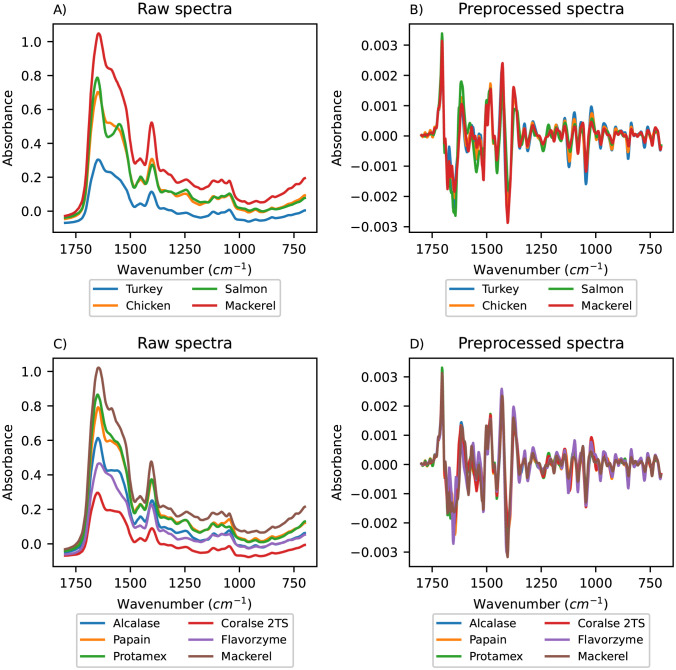
FT-IR spectra of the mean of the samples for each animal and mean of the samples for each enzyme. The mean raw spectra for each animal (A), the mean pre-processed spectra for each animal (B), the mean raw spectra for each enzyme (C) and the mean pre-processed spectra for each enzyme (D).

To gain an understanding of the data, the mean of the samples for each animal and the mean of the samples for each enzyme were plotted. This was done for both the raw data and for the pre-processed data, and the spectra are shown in Fig 2. The enzymes used were Alcalase, Papain, Protamex, Flavorzyme, Corolase 2TS, and additionally some of the mackerel samples were self-hydrolysed (labelled NaN in Fig 2). The data was mean centred (mean of 0) before PLSR and mean centred and standardised (mean of 0, standard deviation of 1) before CNN, RNN and SVM, since this gave the best prediction results for our data set. Using the results obtained with the pre-processing methods giving the best prediction results was chosen in order to get a fair comparison of the methods.

In the same way as for the simulated data set, the raw material data was randomly divided into training and test sets with half of the data set (50%) in each. This split would likely give a good representation of each of the 28 groups in both the training and test data. Determination of the parameters for the global PLSR model and all hierarchical models was done by LOOCV on the training data, prior to the final test set prediction using the independent test set.

### Model parameters

The CNN consisted of one convolutional layer with five filters and a kernel size of 11, the Exponential Linear Unit (Elu) as the activation function, and one max pooling layer. The RNN consisted of two recurrent layers, the first with return sequences activated on 32 nodes, the second on 16 nodes and activation Elu in the recurrent layers and linear activation in the last dense layer. For both CNN and RNN, the networks were trained on 1000 epochs, and the number of epochs used were determined by LOOCV for the raw material data set and 10-fold cross validation for the simulated data on the training sets, for both the global model and the hierarchical models. For SVR we used a grid search to find the local model parameters from linear, radial basis function or sigmoid kernel with the regularisation parameter between 0.0001 and 1000000.

## Results

### Simulation study

The simulated data set was divided into a training (50%) and test (50%) set and applied to HC-PLSR, HC-CNN, HC-RNN and HC-SVR. The performance using FCM for both clustering and classification are shown together with the results from the global models in Table 1. Only FCM was used here, as the clusters were so well-defined that any clustering and classification method would end up with the same solution.

**Table 1:** R^2^-scores for the simulated data set. R^2^-scores from the test set prediction for HC-PLSR, HC-CNN, HC-RNN and HC-SVR for three clusters using FCM. The other three classification methods yielded the same predictions as FCM. “Optimised” refers to the models based on the parameters which gave the highest accuracy after the parameter otimisation for HC-CNN and HC-RNN (described below).

Model	FCM	Global model
HC-PLSR	0.812	0.370
HC-CNN	0.802	0.953
HC-RNN	0.930	0.947
HC-SVR	0.896	0.793
HC-CNN optimised	0.886	0.889
HC-RNN optimised	0.968	0.965

**Fig 3:**
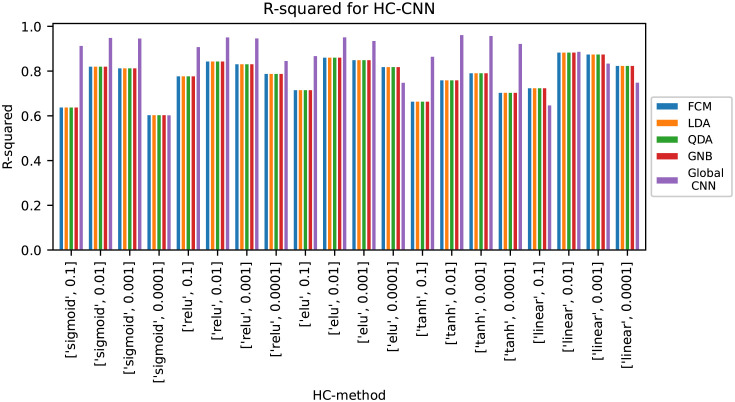
Results from parameter optimisation for HC-CNN with various activation functions and learning rates.

**Fig 4:**
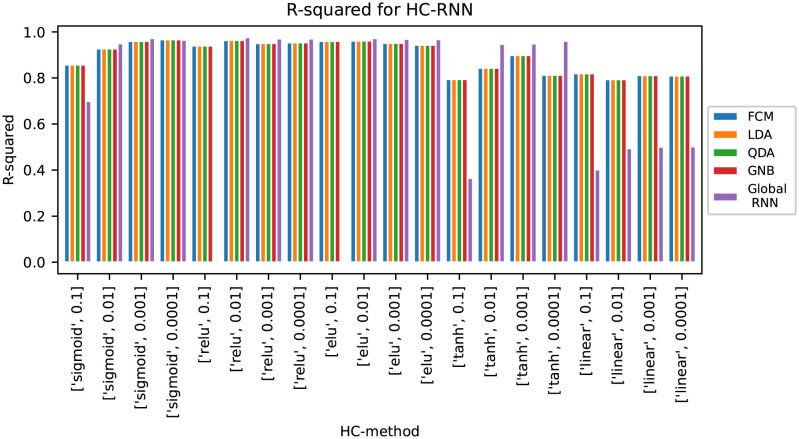
Results from parameter optimisation for HC-RNN with various activation functions and learning rates.

The results in Table 1 show that for PLSR and SVR there is a lot to gain from using the hierarchical modelling versions for this data set, while for CNN and RNN the global models outperformed the hierarchical models. To analyse whether this was due to the specific parameters that were chosen in this study, an additional parameter search was performed to evaluate the effect of using other activation functions and learning rates. The activation functions tested were sigmoid using a logistic curve, ReLU using a ramp function, Elu (the exponential linear units), tanh which uses a hyperbolic tangent and linear activation, while the learning rates were 0.1, 0.01, 0.001 and 0.0001. The results from this parameter search for HC-CNN are shown in Fig 3 and the results for HC-RNN are shown in Fig 4. The results from the parameters giving the highest accuracy for the hierarchical models are shown in Table 1 as “HC-CNN optimised” and “HC-RNN optimised”.

For HC-CNN, the hierarchical models outperformed the global model in 5 out of the 20 tests. The parameters that gave the highest accuracy in the hierarchical models were linear activation and a learning rate of 0.01, but the differences between the accuracy of the global and hierarchical models were relatively small. When using linear activation the hierarchical models performed better overall than the global models. The linear activation has less complexity than the other activation functions, which could make the global models unable to handle the non-linearities in the data, but this is then handled by the local modelling.

For HC-RNN, the hierarchical models outperformed the global model in 9 out of the 20 tests. The parameters that gave the highest accuracy in the hierarchical models were sigmoid activation and a learning rate of 0.0001. For these parameters, the global model performed worse, although not significantly so. For the high learning rates, the global RNN models performed poorly and even failed completely for some activation functions. This indicates that the RNNs need longer time to converge than the CNNs. With linear activation function the hierarchical models performed much better than the global models for all learning rates.

### Raw material data set

**Fig 5:**
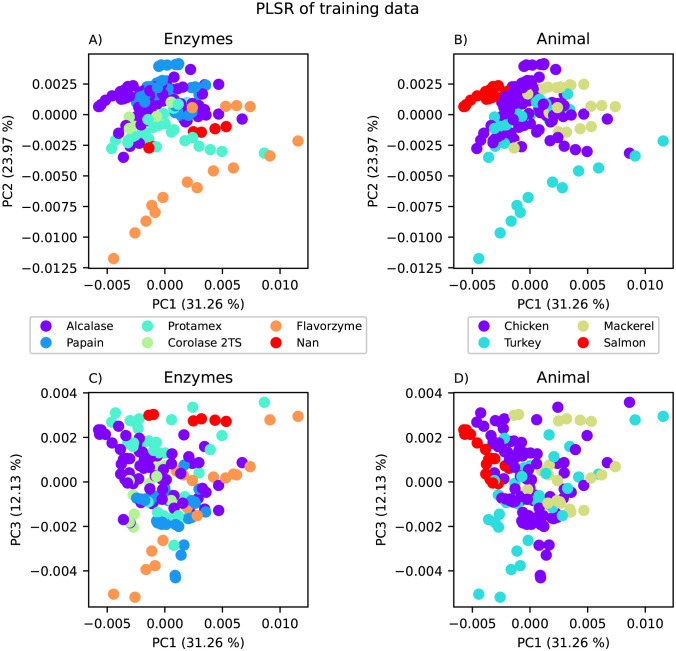
PLSR scoreplot of the raw material data set. PLSR scoreplot where in the plots to the left (A and C) samples are coloured by hydrolysis enzyme, while in the plots to the right (B and D) samples are coloured by animal of origin. The top plots (A and B) show PC1 against PC2, while the lower plots (C and D) show PC1 against PC3.

A global PLSR model was built on the training data from the FT-IR data set, and using LOOCV, the optimal number of components was determined to be 28. However, it was observed that after 5 PCs, the increase in total explained variance was small. Therefore, an additional restraint that each included component should account for more than 1% of the explained variance was added. With this constraint, the optimal number of components was determined to be 11, explaining 93.34% of the variance. A PLSR scoreplot of the first three PC’s is shown in Fig 5 where **A** and **C** are coloured by the enzyme used for hydrolysis while **B** and **D** are coloured by the animal the raw material originated from. The scoreplot shows the distributions of samples and how the data is grouped based on prior knowledge. The first three components explain 67.36% of the variance, however there are no easily separable groups, either for animal or enzymes. This indicates that using local modelling based on cluster analysis instead of prior knowledge might be advantageous for this data set.

#### Optimal number of clusters

To identify the optimal number of clusters to use, models with 2–10 clusters were calibrated using the training set. The training data was divided into a calibration set (50%) and a validation set (50%) where the calibration set was used to calibrate the hierarchical models using LOOCV. The samples in the validation set were then classified and predicted by the respective model. This was done to validate the classification done by FCM, LDA, QDA and NB. This validation was done by test set validation instead of LOOCV, because of the high computational time and demand of running 166 models 166 times. The results are shown in the S1 File. The optimal number of clusters for each method was determined using the maximum of the mean prediction ability (R^2^) over the four classification methods, and confirmed by visual inspection. When using FCM clustering, the optimal number of clusters was determined to be 3 for PLSR and 2 for CNN, RNN and SVR, as all the four classification methods achieved high R^2^ values. For higher numbers of clusters, the classification models were fluctuating, and for HC-PLSR and HC-CNN they even showed a decreasing trend in classification accuracy. Additionally, with the limited amount of samples available, a high number of clusters would mean few samples in each cluster, something that yields poorer predictions/generalisability. The risk of overfitting clearly increases with an increasing number of clusters, so keeping the number of clusters relatively low is generally advisable.

The distributions of samples in the various clusters for all the clustering methods tested are shown in the S1 File along with the figures determining the optimal number of clusters for HAC and SPC.

**Fig 6:**
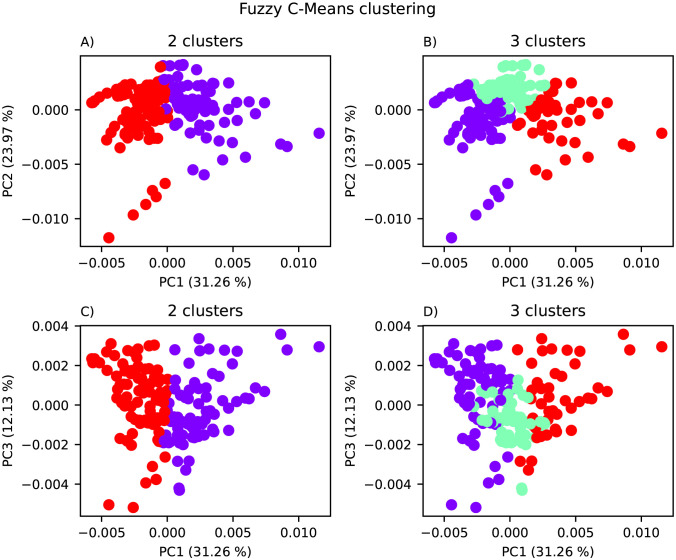
PLSR scoreplot of the FCM results. PLSR scoreplot showing the FCM results using the optimal number of clusters; 2 for SVR, CNN and RNN (A and C) and 3 for PLSR (B and D). The top plots show PC1 against PC2 (A and B) while the bottom plots show PC1 against PC3 (C and D).

To evaluate the properties of the clusters, scoreplots from PLSR with results from FCM using 2 and 3 clusters are shown in Fig 6. For the 2-cluster model, there were 77 samples in cluster 1 and 89 samples in cluster 2. For the 3-cluster model, there were 65 samples in cluster 1, 57 in cluster 2, and 44 in cluster 3. Hence, no clusters with very few samples were found. However, the clusters are not similar to those given by animal type or enzyme from Fig 3.

#### Prediction of the average molecular weight

With the optimal number of clusters determined for HC-PLSR, HC-CNN, HC-RNN and HC-SVR, the hierarchical models were trained and applied on the test set. The performance of the models over the four classification methods is shown in Table 2. The results from the FCM clustering were compared to those from HAC and SPC to evaluate whether a different clustering technique could find more informative clusters. The results obtained with the global models were compared to those obtained with the hierarchical modelling to evaluate the gain of using local modelling. FCM was unable to classify samples when using HAC and SPC, as the FCM did not have the possibility to train on labels, and was therefore not able to assimilate the clusters from the other two methods.

**Table 2:** R^2^-scores for the raw material data set. R^2^-scores from the test set prediction for HC-PLSR, HC-CNN, HC-RNN and HC-SVR for their optimal number of clusters using FCM, HAC and SPC.

Model	FCM	LDA	QDA	NB	# clusters	Global model	Clustering
HC-PLSR	0.696	0.654	0.691	0.804	3	0.797	FCM
HC-CNN	0.748	0.737	0.759	0.732	2	0.795	FCM
HC-RNN	0.810	0.798	0.814	0.818	2	0.836	FCM
HC-SVR	0.850	0.831	0.871	0.812	2	0.871	FCM
HC-PLSR	-	0.593	0.641	0.710	2	0.797	HAC
HC-CNN	-	0.787	0.795	0.786	2	0.795	HAC
HC-RNN	-	0.781	0.779	0.789	2	0.836	HAC
HC-SVR	-	0.858	0.879	0.843	2	0.871	HAC
HC-PLSR	-	0.824	0.844	0.736	4	0.797	SPC
HC-CNN	-	0.752	0.776	0.771	2	0.795	SPC
HC-RNN	-	0.799	0.808	0.823	2	0.836	SPC
HC-SVR	-	0.860	0.871	0.848	2	0.871	SPC

Overall, our results show that for this data set, both the global PLSR and the HC-PLSR perform poorly. Especially for the FCM clustering, an increase in prediction ability is observed compared to HC-PLSR for the local deep learning methods for all classification methods except NB. During inspection of the clusters in HAC and SPC, overlapping clusters and boundaries were observed (shown in the S1 File) which can make the clustering more inaccurate. Additionally, the clusters did not make much sense compared to the groupings observed in Fig 5 and these clustering methods were therefore not evaluated further. For the NN models, CNN gave lower R^2^-values than the global PLSR both for the global CNN and for HC-CNN. Hence, CNN was not an optimal method for this data set. RNN performed overall well, although the global model performed better than the HC-RNNs. The best models achieved for this data set were obtained using SVR, both in the global and hierarchical modelling. This is a strong indication of the presence of some non-linearities that HC-PLSR could not account for and that this data set requires non-linear methods to be modelled optimally.

#### Feature importance

**Fig 7:**
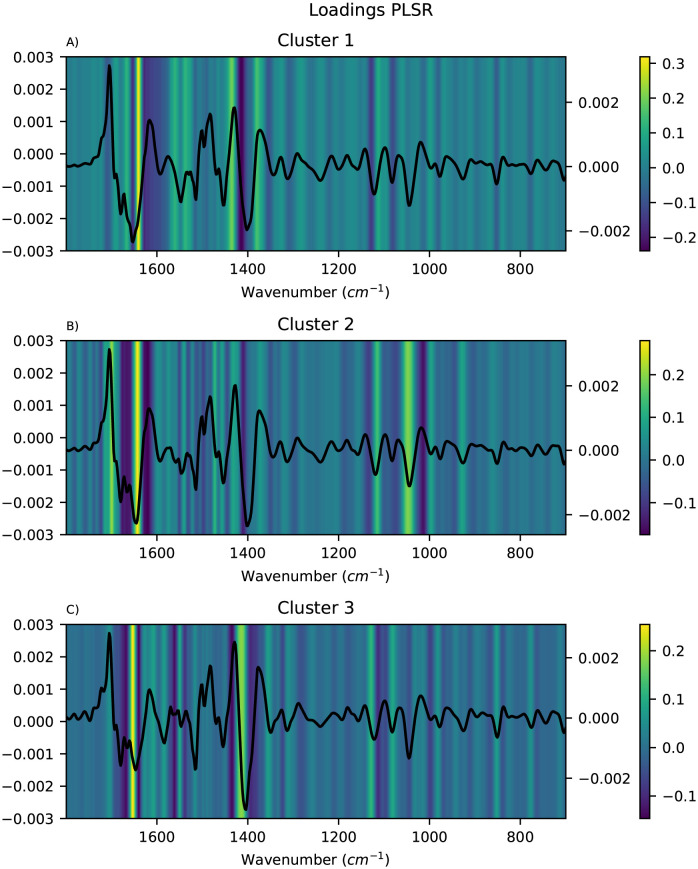
Visualisation of the feature importance for HC-PLSR. Visualisation of the feature importance from the loadings of the first PLS component for the HC-PLSR with three clusters found using FCM, with the mean spectra of the samples in the corresponding cluster overlayed. Important features are coloured yellow and unimportant are blue.

**Fig 8:**
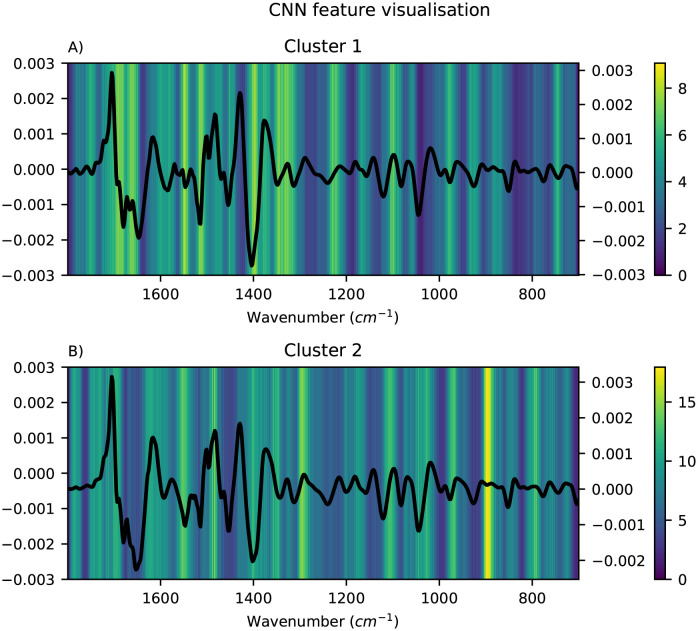
Visualisation of the feature importance for HC-CNN. Visualisation of the feature importance for the HC-CNN with two clusters found using FCM, cluster 1 in A and cluster 2 in B, with the mean spectra of the samples in the corresponding cluster overlayed. Important features are coloured yellow and unimportant are blue.

**Fig 9:**
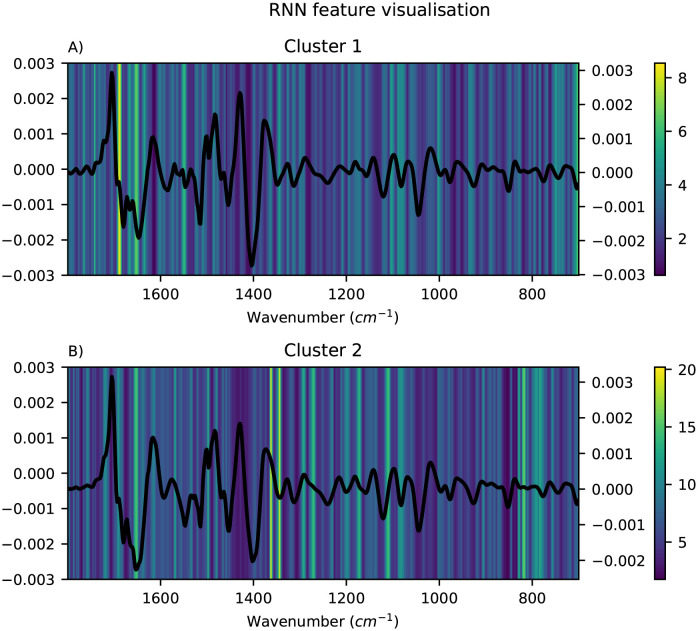
Visualisation of the feature importance for HC-RNN. Visualisation of the feature importance for the HC-RNN with two clusters found using FCM, cluster 1 in A and cluster 2 in B, with the mean spectra of the samples in the corresponding cluster overlayed. Important features are coloured yellow and unimportant are blue.

**Fig 10:**
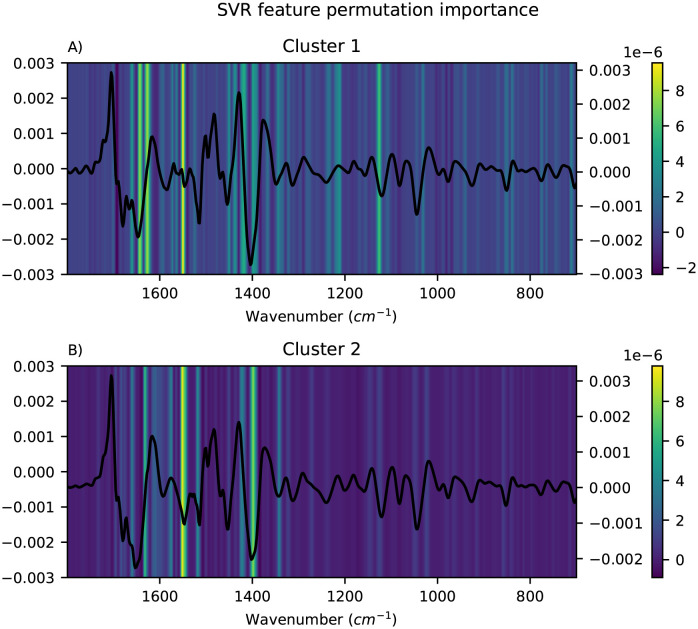
Visualisation of the feature importance for HC-SVR. Visualisation of the feature importance for the HC-SVR with two clusters found using FCM, cluster 1 in A and cluster 2 in B, with the mean spectra of the samples in the corresponding cluster overlayed. Important features are coloured yellow and unimportant are blue.

The important features for each of the local modelling methods were visualised as heatmaps and the mean pre-processed spectra for the corresponding cluster were overlayed to simplify the interpretation. Only the results obtained using FCM clustering are shown here, since the alternative clustering methods did not give more meaningful clusters (see S1 File). For HC-PLSR, the important features were visualised using the loadings for each of the local models, and the results are shown in Fig 7. For each of the local models in HC-CNN and HC-RNN, VarGrad was applied to identify the important features. The results for CNN are shown in Fig 8, where the important features are yellow and the unimportant are blue. For RNN, the results are shown in Fig 9. Lastly, for HC-SVR, the important features were obtained using permutation feature importance for each local model, and the results are shown in Fig 10.

In dry-film FTIR spectra, prominent hydrolysis markers are ∼1400 cm^-1^ corresponding to carboxylate (COO^-^), 1516 cm^-1^ to ammonia (NH^+^_3_), ∼1550 cm^-1^ to amide II and ∼1650 cm^-1^ corresponding to amide I [4, 43, 44].

From Figs 8 and 9, it is easy to spot the peak corresponding to the carboxylate-group at ∼1400 cm^-1^, and in the HC-CNNs, this peak has a light yellow colour indicating that it is given weight by the networks in both clusters, but slightly more in cluster 1. For the HC-SVRs, this peak also has a lighter yellow colour for both clusters, and it is lighter and therefore of higher importance in cluster 2. For the HC-PLSR models, this peak is lightest in cluster 3.

The spectra show that the samples with a large effect from the ammonia peak at 1516 cm^-1^ are placed in cluster 1, and in HC-CNN and HC-RNN, this peak is deemed important. In HC-SVR, this peak is of higher importance in cluster 2. In HC-PLSR these samples are placed in cluster 3, which is also where this peak is most important.

The amide II groups at ∼1550 cm^-1^ show importance in cluster 1 for all methods and in cluster 2 for HC-SVR and HC-PLSR. Lastly, the samples with a larger peak at ∼1650 cm^-1^, are placed in cluster 1, and this peak seems to have a higher importance in cluster 1 compared to cluster 2 for HC-CNN, HC-RNN and HC-SVM, while for HC-PLSR this peak is important in all three clusters.

The models also find peaks of importance at ∼1350 cm^-1^ and at ∼1100 cm^-1^, and cluster 2 in HC-CNN shows a strong importance of a peak at ∼900 cm^-1^. ∼1350 cm^-1^ could possibly correspond to nitro groups, ∼900 cm^-1^ could be aromatic rings and ∼1100 cm^-1^ could be ether absorption [45]. All could be heteroatom subtitutions (for example oxygen, sulphur or nitrogen groups) as well. These three areas are in the fingerprint region, between 1400–700 cm^-1^, which usually contains a large number of peaks, which is also noticeable by how the hierarchical models all highlight different parts of this area. Additionally, peaks in the fingerprint region often correspond to asymmetric stretching vibrations which also could explain the peaks at ∼1350, ∼1100 and ∼900 cm^-1^. For HC-SVR, the peak at ∼1100 cm^-1^ is only found to be important in the local model made in cluster 1.

As the global models contain all the information in the data set and have to model all of it, they become more general and do not manage to capture the differences that lie in the sub-structures of the data. Such variations will thus be overshadowed in the global model, but can be captured in the local models. This is illustrated by the loadingplot for the global PLSR model shown in S1 File, which gives a more overall picture of the feature importances, and fails to identify all the nuances that lie in the regional differences shown in Fig 7. The HC set-up can be regarded as a sensitivity analysis, as the ability to interpret the NN models is increased using the feature importance visualisation combined with inspection of the latent variables from PLSR, which the clustering is based on. Further updates of the model to decrease uncertainties could also be implemented in the model in the future, however this also increases computation time, which is already high due to the size of the data set.

## Discussion

Our results showed that HC-CNN (with optimised parameters), HC-RNN and HC-SVR yielded better predictions than HC-PLSR for the simulated data set, while for the raw material data set only SVR (both hierarchical and global SVR) performed better than HC-PLSR. This demonstrates that the linearity in PLSR represents a disadvantage when the data is not locally linear in structure (as for the simulated data set), and hence HC-PLSR is not performing as well as the deep learning-based hierarchical methods. For the raw materials data set, HC-PLSR also needed a higher number of clusters than the other methods to handle non-linearities in the data, since this lies intrinsic in the other methods.

The simulation study proved the proficiency of the expansion of local modelling to deep learning; after the parameter search, HC-CNN, HC-RNN and HC-SVR all outperformed HC-PLSR. The parameter search performed for HC-CNN and HC-RNN could also be expanded further, although this would be more time consuming and was therefore not prioritised in this study. For HC-PLSR and HC-SVR, the advantages of using local modelling were clearly shown by the prediction abilities, however for HC-CNN and HC-RNN, the results for the global and hierarchical models were quite similar, and the global models were often slightly better. This is probably due to that CNN and RNN are methods developed to model patterns in data, and the networks are likely able to create something similar to local modelling through the optimisations of the weights. Moreover, the number of observations used to train the networks is naturally higher for the global models. Hence for these methods, the gain in using hierarchical deep learning lies in the interpretation, not in the predictive power.

The visualisation of the feature importance for the hierarchical models of the raw material data set showed that the models evaluated different features as important between the clusters. This illustrates the benefits of using the local modelling when it comes to interpretation. Moreover, the latent variables from the global PLSR model that the clustering is based on, provide the opportunity to visualise covariance patterns both between ***X*** and ***Y***, and within the ***X***- and ***Y***- matrices. This can be very useful when interpreting the complex neural network-based models. The latent variables give an overview of the properties of the different clusters, which is useful for understanding the underlying reasons for differences in feature importance between the clusters.

Local modelling allows for simpler models, models with a low number of PCs, few convolutional layers etc. as well as increased prediction ability and interpretability. The hierarchical methods can handle unlimited numbers of clusters, the limitations only lie in the number of samples. However, in order to avoid overfitting, the number of clusters used should be kept relatively low. In HC-PLSR, using a low number of PCs is also beneficial for the clustering, creating simpler clusters and subsequent models. With either method, one should strive to keep the model complexity as low as possible, but without sacrificing prediction ability, both to limit the risk of overfitting and to ease interpretation. As opposed to many previously published local modelling procedures, the local modelling described in this paper is fully automatic, i.e. no prior information about groups in the samples is required. This is an advantage when the structures in the data are unknown, which is often the case.

For the deep learning models, a disadvantage is that the data set has to be large enough for both training and validation. Due to the risk of overfitting with deep neural networks, having a large enough validation and test set is crucial. It is therefore important to ensure that the number of observations in the local models is high enough for sufficient validation. Small data sets can result in unstable networks, especially if the number of features is substantially larger than the number of samples. Therefore, to evaluate the robustness of the method in various scenarios, one data set which had more samples than features (simulated data set) and one with more features than samples (raw material data set) was selected. The results showed that the HC framework were suitable for both scenarios and also yields relatively simple models for both data sets.

The methodology presented here can therefore in principle handle any kind of data. In data consisting of chemical spectra, parts of the data are often connected by for instance homologeous series, isotope peaks, adjacent groups or chemical bonds, which are essential for the identification and analysis of chemical structures. Models which can easily detect these patterns even in data sets with large differences between the samples, are of great value. The comparison of the hierarchical models to the global models can illuminate structures in the data which the global model is not able to capture and the hierarchical models can also be used to identify problem areas in the data if any of the clusters yield significantly poorer prediction abilities than the remaining ones.

## Conclusion

In this study, the previously published HC-PLSR procedure was extended to deep learning and SVR, in order to allow for modelling of data that is not locally linear, as well as increase the interpretability of deep learning models through identification of regional differences in feature importance. Analysis of a highly non-linear simulated data set indicated that in cases where the data can not be split into clusters having a linear data structure, the deep learning-based models have better prediction abilities than HC-PLSR. Application of the developed methodology to a spectroscopic data set identified differences in feature importance between different regions of the data space that were useful for model interpretation. Expanding the HC-PLSR framework to deep learning provides the opportunity to combine the predictive power of deep learning for highly non-linear data, with the interpretability provided by the decomposition into a lower-dimensional space of latent variables from PLSR and local cluster-based modelling. As the clustering in the hierarchical modelling is done on the latent variables from a global PLSR model, useful for identifying the underlying reasons for differences in feature importance between the clusters, this provides advantages even in cases where the prediction ability of the PLSR model is moderate.

## Supporting information

S1 File Overview of additional parameters tested for the hierarchical models. Includes additional clustering results, optimal number of clusters, distribution of simulated data and important feature for global PLSR model.
(PDF)

## References

[1] Wold S, Martens H, Wold H. The multivariate calibration problem in chemistry solved by the PLS method. In: Matrix Pencils. No. 973 in Lecture Notes in Mathematics. Berlin, Heidelberg: Springer; 1983. p. 286–293. Available from: https://link.springer.com/chapter/10.1007/BFb0062108.

[2] Eriksson L, Trygg J, Wold S. PLS-trees^®^, a top-down clustering approach. Journal of Chemometrics. 2009;23(11):569–580. doi: 10.1002/cem.1254

[3] Eriksson L, Toft M, Johansson E, Wold S, Trygg J. Separating Y-predictive and Y-orthogonal variation in multi-block spectral data. Journal of Chemometrics. 2006;20(8-10):352–361. doi: 10.1002/cem.1007

[4] Kristoffersen KA, Liland KH, Böcker U, Wubshet SG, Lindberg D, Horn SJ, et al. FTIR-based hierarchical modeling for prediction of average molecular weights of protein hydrolysates. Talanta. 2019;205:120084. doi: 10.1016/j.talanta.2019.06.084 31450429

[5] Lindström A, Pettersson F, Almqvist F, Berglund A, Kihlberg J, Linusson A. Hierarchical PLS Modeling for Predicting the Binding of a Comprehensive Set of Structurally Diverse Protein–Ligand Complexes. Journal of Chemical Information and Modeling. 2006;46(3):1154–1167. doi: 10.1021/ci050323k 16711735

[6] Wold S, Kettaneh N, Tjessem K. Hierarchical multiblock PLS and PC models for easier model interpretation and as an alternative to variable selection. Journal of Chemometrics. 1996;10:463–482. doi: 10.1002/(SICI)1099-128X(199609)10:5/6<463::AID-CEM445>3.0.CO;2-L

[7] Wold S, Berglund A, Kettaneh N. New and old trends in chemometrics. How to deal with the increasing data volumes in R&D&P (research, development and production)—with examples from pharmaceutical research and process modeling. Journal of Chemometrics: Special Issue: Proceedings of the 7th Scandinavian Symposium on Chemometrics. 2002;16(8-10):377–386. doi: 10.1002/cem.746

[8] Bevilacqua M, Marini F. Local classification: Locally weighted–partial least squares-discriminant analysis (LW–PLS-DA). Analytica Chimica Acta. 2014;838:20–30. doi: 10.1016/j.aca.2014.05.057 25064239

[9] Tøndel K, Indahl UG, Gjuvsland AB, Vik JO, Hunter P, Omholt SW, et al. Hierarchical Cluster-based Partial Least Squares Regression (HC-PLSR) is an efficient tool for metamodelling of nonlinear dynamic models. BMC Systems Biology. 2011;5:Article 90. doi: 10.1186/1752-0509-5-90 21627852 PMC3127793

[10] Tøndel K, Indahl UG, Gjuvsland AB, Omholt SW, Martens H. Multi-way metamodelling facilitates insight into the complex input-output maps of nonlinear dynamic models. BMC Systems Biology. 2012;6:88. doi: 10.1186/1752-0509-6-88 22818032 PMC3483253

[11] Gath I, Geva A B. Unsupervised optimal fuzzy clustering. IEEE Transactions on Pattern Analysis and Machine Intelligence. 1989;11(7):773–780. doi: 10.1109/34.192473

[12] Frigui H, Krishnapuram R. A robust competitive clustering algorithm with applications in computer vision. IEEE Transactions on Pattern Analysis and Machine Intelligence. 1999;21(5):450–465. doi: 10.1109/34.765656

[13] Dunn JC. A Fuzzy Relative of the ISODATA Process and Its Use in Detecting Compact Well-Separated Clusters. Journal of Cybernetics. 1973;3(3):32–57. doi: 10.1080/01969727308546046

[14] Bezdek JC. Pattern Recognition with Fuzzy Objective Function Algorithms. 1st ed. Advanced Applications in Pattern Recognition. Springer US; 1981. Available from: https://www.springer.com/gp/book/9781475704525.

[15] von Luxburg U. A tutorial on spectral clustering. Statistics and Computing. 2007;17:395–416. doi: 10.1007/s11222-007-9033-z

[16] Nielsen F. Hierarchical Clustering. In: Introduction to HPC with MPI for Data Science. 1st ed. Undergraduate Topics in Computer Science. Springer; 2016. p. 195–211. Available from: https://www.researchgate.net/publication/314700681_Hierarchical_Clustering.

[17] Wold S, Ruhe A, Wold H, Dunn WJ III. The Collinearity Problem in Linear Regression. The Partial Least Squares (PLS) Approach to Generalized Inverses. SIAM Journal on Scientific and Statistical Computing. 1984;5(3):735–743. doi: 10.1137/0905052

[18] Martens H, Næs T. Multivariate Calibration. 1st ed. Chichester: Wiley; 1992.

[19] Wold S, Sjöström M, Eriksson L. PLS-regression: a basic tool of chemometrics. Chemometrics and Intelligent Laboratory Systems. 2001;58(2):109–130. doi: 10.1016/S0169-7439(01)00155-1

[20] LeCun Y, Bottou L, Bengio Y, Haffner P. Gradient-based learning applied to document recognition. Proceedings of the IEEE. 1998;86(11):2278–2324. doi: 10.1109/5.726791

[21] LeCun Y, Bengio Y, Hinton G. Deep learning. Nature. 2015;521:436–444. doi: 10.1038/nature14539 26017442

[22] Mandic DP, Chambers JA. Recurrent Neural Networks for Prediction: Learning Algorithms, Architectures and Stability. Wiley; 2001. Available from: https://onlinelibrary.wiley.com/doi/book/10.1002/047084535X.

[23] Sarker IH. Deep Learning: A Comprehensive Overview on Techniques, Taxonomy, Applications and Research Directions. SN Computer Science. 2021;2:420. doi: 10.1007/s42979-021-00815-1 34426802 PMC8372231

[24] Drucker H, Burges CJC, Kaufman L, Smola A, Vapnik V. Support vector regression machines. In: Advances in Neural Information Processing Systems 9. Cambridge, MA: MIT Press; 1997.

[25] Vapnik V, S G, A S. Support vector method for function approximation, regression estimation, and signal processing. In: Advances in Neural Information Process Systems 9. 1st ed. Cambridge, MA: MIT Press; 1997. p. 281–287.

[26] Burges CJC. A Tutorial on Support Vector Machines for Pattern Recognition. Data Mining and Knowledge Discovery. 1998;2:121–167. doi: 10.1023/A:1009715923555

[27] Dias MLD. fuzzy-c-means: An implementation of Fuzzy C-means clustering algorithm.; 2019. Available from: https://git.io/fuzzy-c-means.

[28] McLachlan GJ. Discriminant Analysis and Statistical Pattern Recognition. 1st ed. Wiley Series in Probability and Statistics. Wiley-Interscience; 1992. Available from: https://onlinelibrary.wiley.com/doi/book/10.1002/0471725293.

[29] Hastie T, Friedman J, Tibshirani R. The Elements of Statistical Learning: Data Mining, Inference, and Prediction. 1st ed. Springer Series in Statistics. New York, NY: Springer; 2001. Available from: https://link.springer.com/book/10.1007/978-0-387-21606-5.

[30] Adebayo J, Gilmer J, Goodfellow I, Kim B. Local Explanation Methods for Deep Neural Networks Lack Sensitivity to Parameter Values. arXiv. 2018; p. 1810.03307. doi: 10.48550/arXiv.1810.03307

[31] Adebayo J, Gilmer J, Muelly M, Goodfellow I, Hardt M, Kim B. Sanity Checks for Saliency Maps. In: Advances in Neural Information Processing Systems 31. Montréal, Canada; 2018. p. 9505–9515. Available from: https://proceedings.neurips.cc/paper/2018/file/294a8ed24b1ad22ec2e7efea049b8737-Paper.pdf.

[32] Hooker S, Erhan D, Kindermans PJ, Kim B. A Benchmark for Interpretability Methods in Deep Neural Networks. In: Advances in Neural Information Processing Systems. vol. 32. Vancouver, Canada: Curran Associates, Inc.; 2019. Available from: https://proceedings.neurips.cc/paper/2019/hash/fe4b8556000d0f0cae99daa5c5c5a410-Abstract.html.

[33] Jenul A, Schrunner S, Huynh BN, Helin R, Futsæther CM, Liland KH, et al. Ranking Feature-Block Importance in Artificial Multiblock Neural Networks. In: Artificial Neural Networks and Machine Learning. vol. 13532 of Lecture Notes in Computer Science. Bristol, UK: Springer, Cham; 2022. Available from: https://link.springer.com/chapter/10.1007/978-3-031-15937-4_14.

[34] Breiman L. Random Forests. Machine Learning. 2001;45:5–32. doi: 10.1023/A:1017934522171

[35] Postma GJ, Krooshof PWT, Buydens LMC. Opening the kernel of kernel partial least squares and support vector machines. Analytica Chimica Acta. 2011;705(1-2):123–134. doi: 10.1016/j.aca.2011.04.025 21962355

[36] Smolinska A, Blanchet L, Coulier L, Ampt KAM, Luider T, Hintzen Rogier Q, et al. Interpretation and Visualization of Non-Linear Data Fusion in Kernel Space: Study on Metabolomic Characterization of Progression of Multiple Sclerosis. PLOS ONE. 2012;7(6):e37163. doi: 10.1371/journal.pone.0038163 22715376 PMC3371049

[37] Vitale R, Noord OEd, Ferrer A. A kernel-based approach for fault diagnosis in batch processes. Journal of Chemometrics. 2014;28(8):S697–S707. doi: 10.1002/cem.2629

[38] Vitale R, Palací-López D, Kerkenaar HHM, Postma GJ, Buydens LMC, Ferrer A. Kernel-Partial Least Squares regression coupled to pseudo-sample trajectories for the analysis of mixture designs of experiments. Chemometrics and Intelligent Laboratory Systems. 2018;175:37–46. doi: 10.1016/j.chemolab.2018.02.002

[39] Friedman j. Multivariate adaptive regression splines. The Annal of Statistics. 1991;19(1):1–67. doi: 10.1214/aos/1176347973

[40] Pedregosa F, Varoquaux G, Gramfort A, Michel V, Thirion B, Grisel O, et al. Scikit-learn: Machine Learning in Python. Journal of Machine Learning Research. 2011;12(85):2825–2830.

[41] Savitzky A, Golay MJE. Smoothing and Differentiation of Data by Simplified Least Squares Procedures. Analytical Chemistry. 1964;36(8):1627–1639. doi: 10.1021/ac60214a047

[42] Martens H, Stark E. Extended multiplicative signal correction and spectral interference subtraction: New preprocessing methods for near infrared spectroscopy. Journal of Pharmaceutical and Biomedical Analysis. 1991;9(8):625–635. doi: 10.1016/0731-7085(91)80188-F 1790182

[43] Böcker U, Wubshet SG, Lindberg D, Afseth NK. Fourier-transform infrared spectroscopy for characterization of protein chain reductions in enzymatic reactions. Analyst. 2017;142(15):2812–2818. doi: 10.1039/C7AN00488E 28686252

[44] Wubshet SG, Måge I, Böcker U, Lindberg D, Knutsen SH, Rieder A, et al. Fourier-transform infrared spectroscopy for characterization of protein chain reductions in enzymatic reactions. Analytical Methods. 2017;9(29):4247–4254. doi: 10.1039/C7AY00865A 28686252

[45] Williams D, Fleming I. Spectroscopic methods in organic chemistry. 6th ed. UK: McGraw-Hill Education; 2008. Available from: https://link.springer.com/book/10.1007/978-3-030-18252-6.

