# Supplementary material for: Increased interpretation of deep learning models using hierarchical cluster-based modelling
Source: PLoS One. 2023 Dec 7;18(12):e0295251. doi: 10.1371/journal.pone.0295251 (PMC10703235; doi:10.1371/journal.pone.0295251)
Supplement: S1 File — Includes additional clustering results, optimal number of clusters, distribution of simulated data and important feature for global PLSR model. (PDF) [file pone.0295251.s001.pdf]

Supporting information

Hierarchical cluster-based deep learning

Elise Lunde Gjelsvik[1], Kristin Tøndel[1*]
[1]Norwegian University of Life Sciences, Faculty for Science and Technology, Ås, Norway
[*]kristin.tondel@nmbu.no

Several different tests were performed while building the HC methods. They are presented in Table S1.

Table S1 Table of different things that were tested during the development of the HC methods and the resulting description of why they were not included in the final model.

Alterations	Information	Result
Turkey samples were removed	72 out of 332 samples	The turkey samples are very different from many of the other samples. Removing them from the model yields a higher accuracy for both global and local models. However, the aim of doing local modelling is to capture this type of variation into different clusters where they can be modelled separately. Therefore, removing them would contradict the point of local modelling.
Different clustering methods	DBSCAN, K-means	DBSCAN is not able to find clusters from this data set, even after a search of all possible parameter combinations. K-means does not yield more sensible clusters than the other methods.
Polynomial PLSR	2. degree polynomial	Polynomial PLSR is more computationally demanding and did not yield a better prediction than regular PLSR
Long short-term memory (LSTM)	HC-LSTM	The LSTM networks were very unstable with poor prediction abilities.
Deeper networks	Additional convolutional layers in the CNN and recurrent layers in the RNN	With deeper networks, interpretability is lost. Additionally, the data set does not contain many samples and could therefore easily overfit with many and large layers. Therefore, small networks are suitable for this data set. However, the global models

		were tested with more layers and the results are shown in table S2.
FCM as classifier for the other clustering methods	FCM for classifications	The FCM package does not have the possibility to train with the labels and does not learn enough from the data to be used solely as a classification method.
Standardisation before PLSR		For this data set, standardisation before PLSR decreased the prediction ability of the models, and PLSR was unable to find the important variation in the data yielding poor prediction.

Table S2 Table of R^2 for additional convolutional layers for CNN and recurrent layers for RNN for the global model

No. layers	R^2 CNN	R^2 RNN
1	0.795	-
2	0.824	0.836
3	0.862	0.847
4	0.823	0.846
5	-	0.840

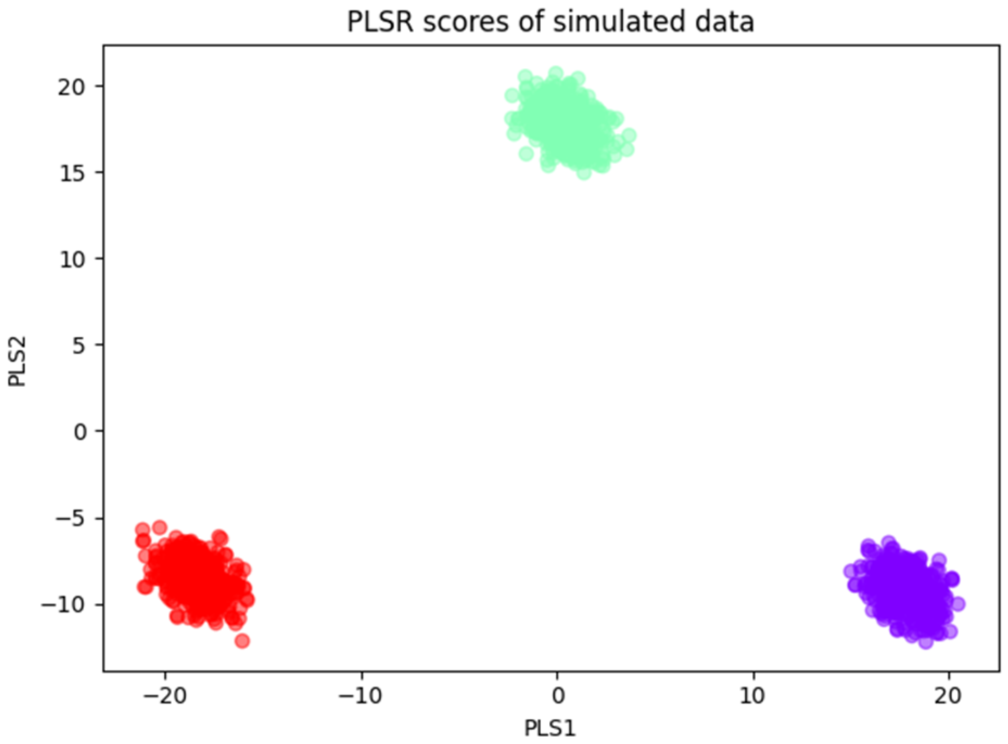

Figure S1 PLSR scores of the simulated data showing the three defined clusters created by the make_friedman and make_blobs functions.

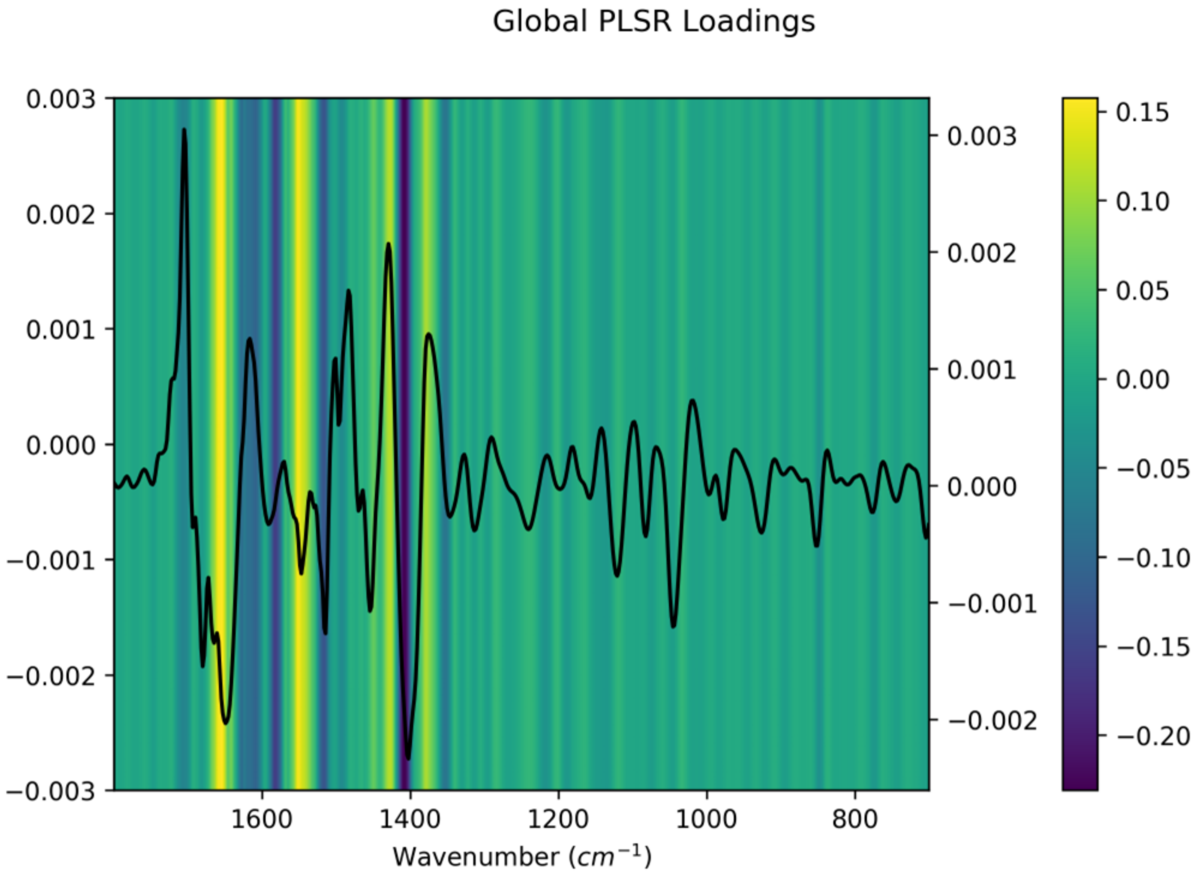

Figure S2 PLSR loadings for the global PLSR model on the training set.

The distributions of samples in clusters 2-10 for all clustering methods evaluated are shown in Figures S3-S7.

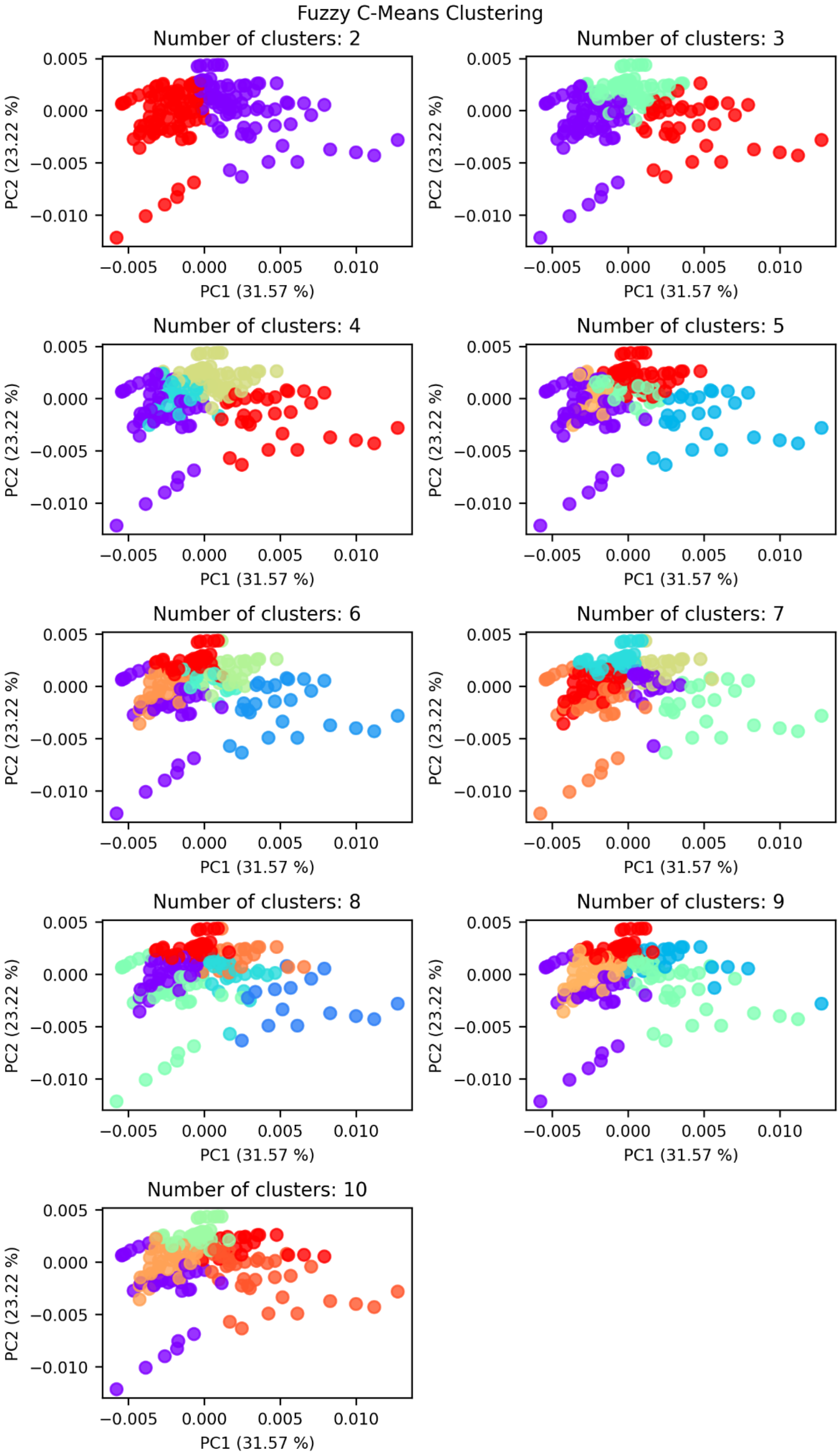

Figure S3 Distribution of samples in cluster 2-10 for Fuzzy C-Means clustering.

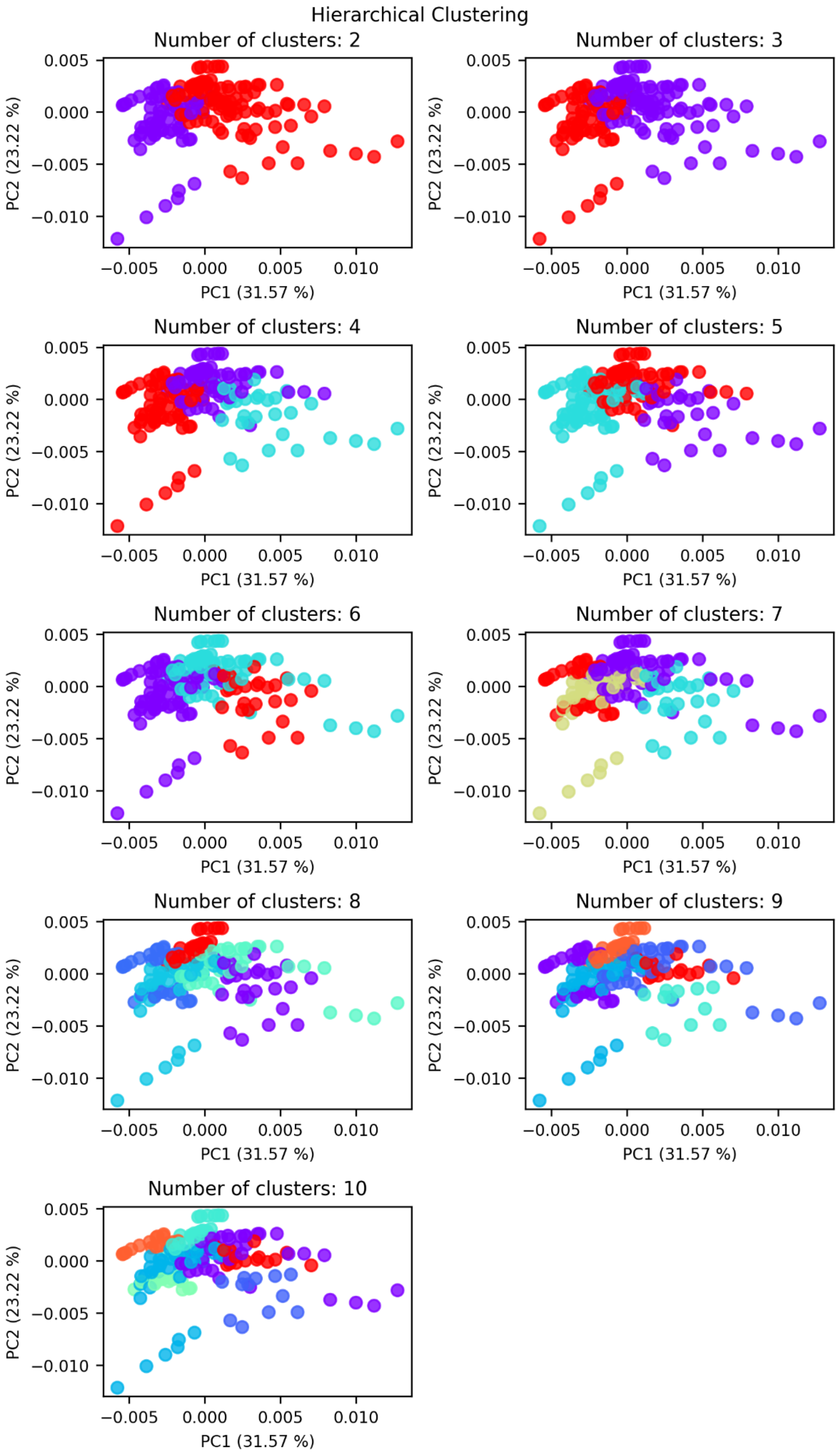

Figure S4 Distribution of samples in clusters 2-10 for Hierarchical Agglomerative clustering.

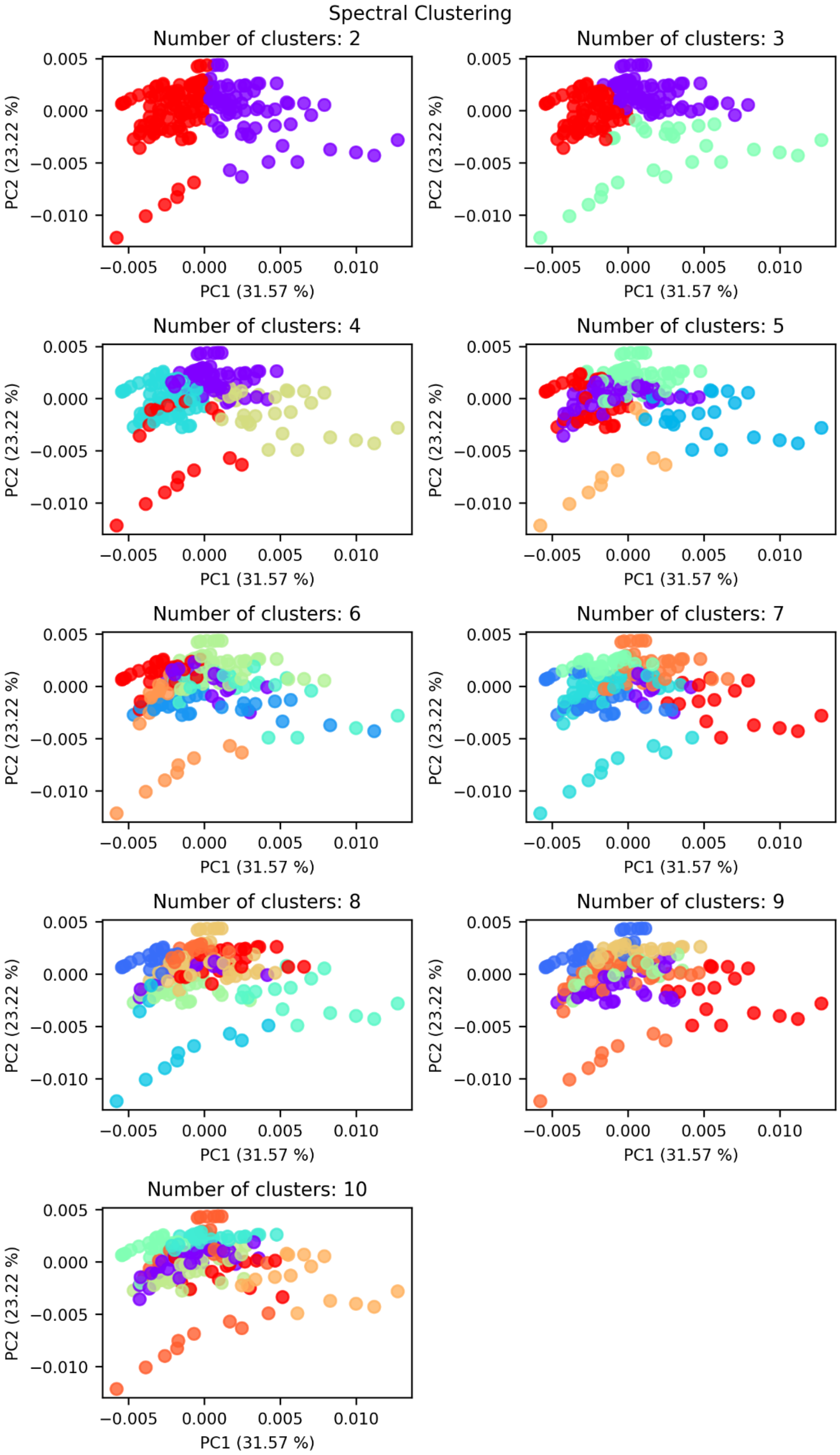

Figure S5 Distribution of samples in cluster 2-10 for Spectral clustering.

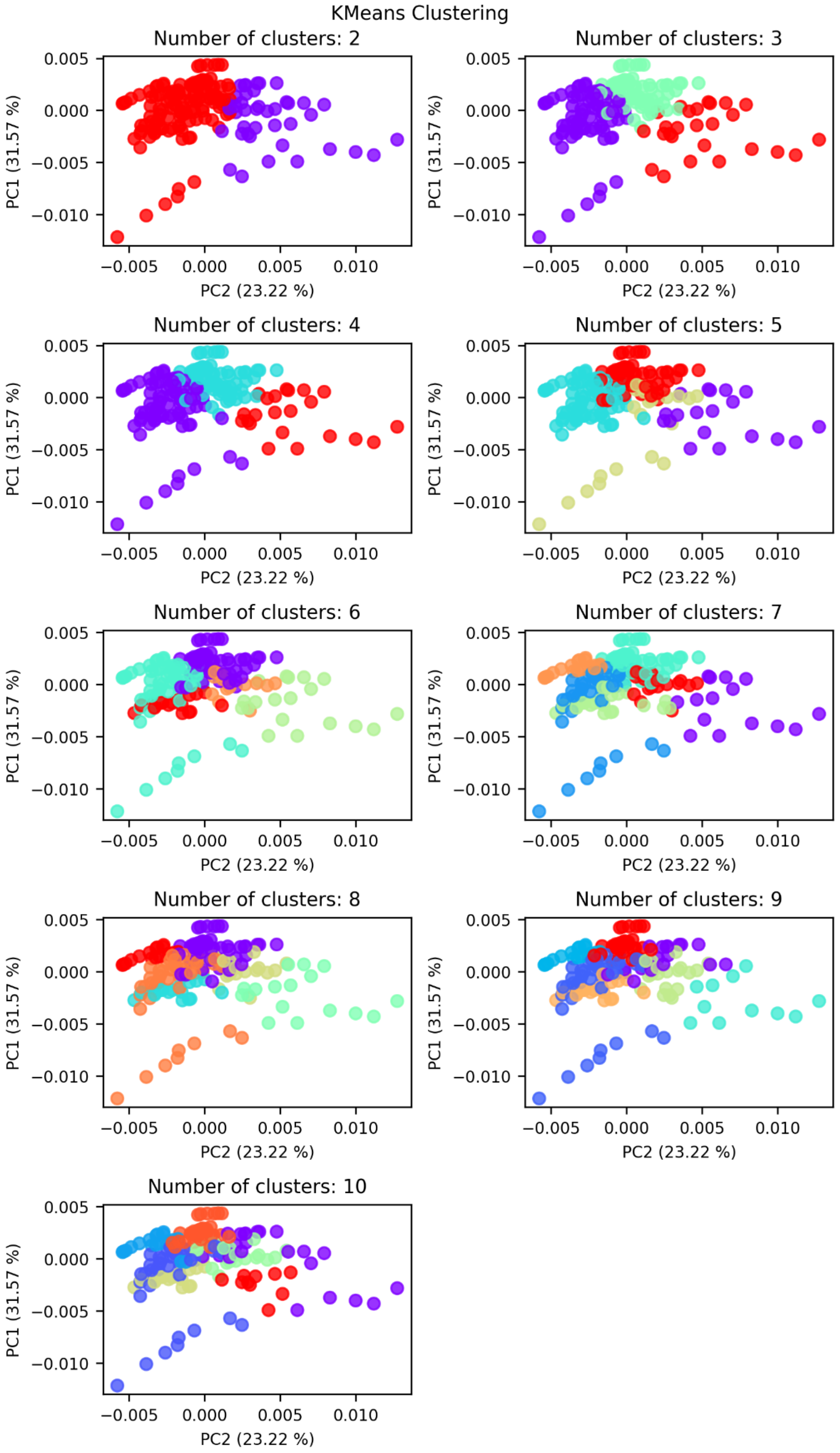

Figure S6 Distribution of samples in cluster 2-10 for K-means clustering.

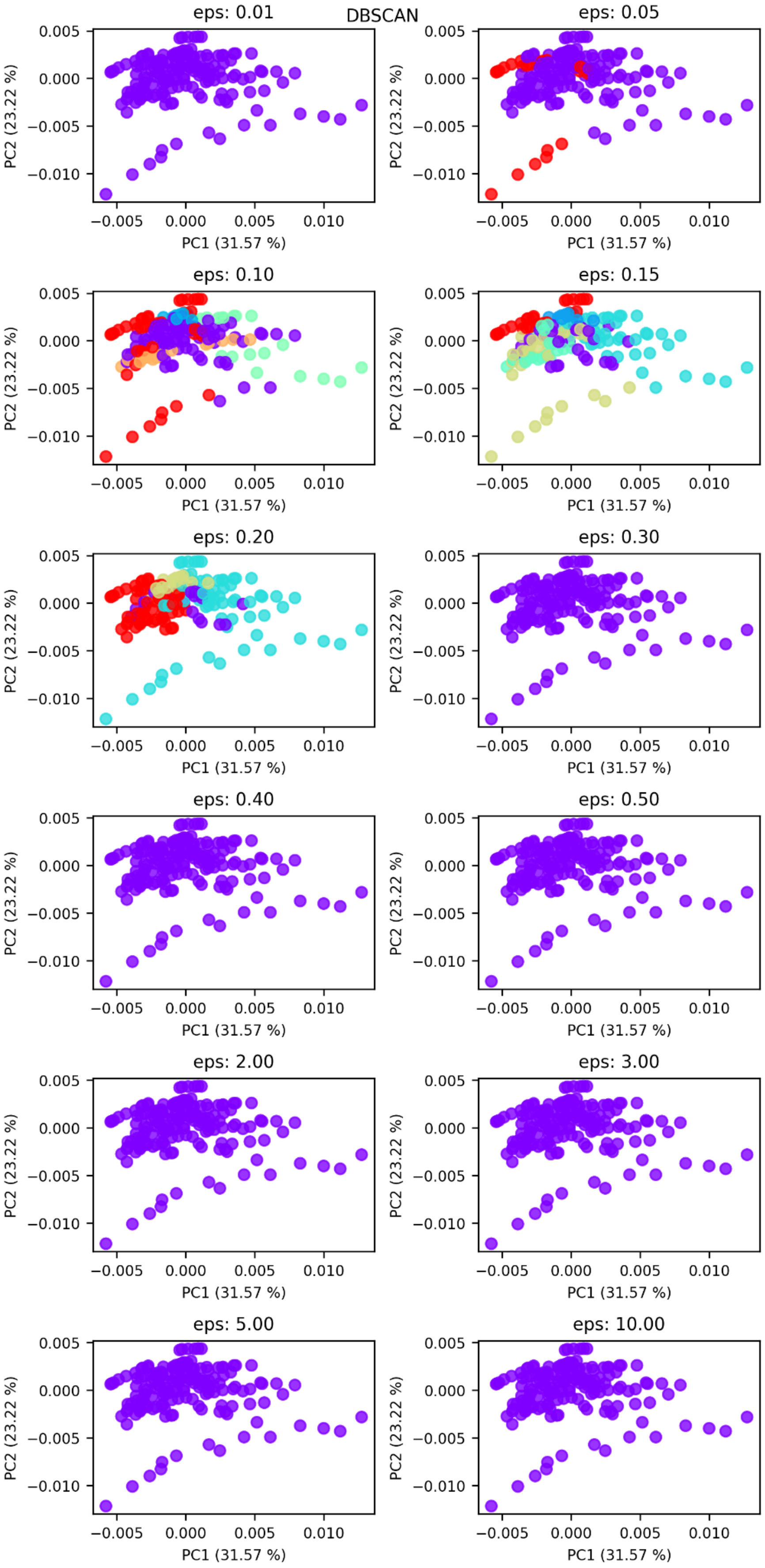

Figure S7 Distribution of samples in clusters 2-10 for DBSCAN.

The optimal number of clusters for Hierarchical Agglomerative clustering and Spectral clustering was determined based on Figures S8-S10.

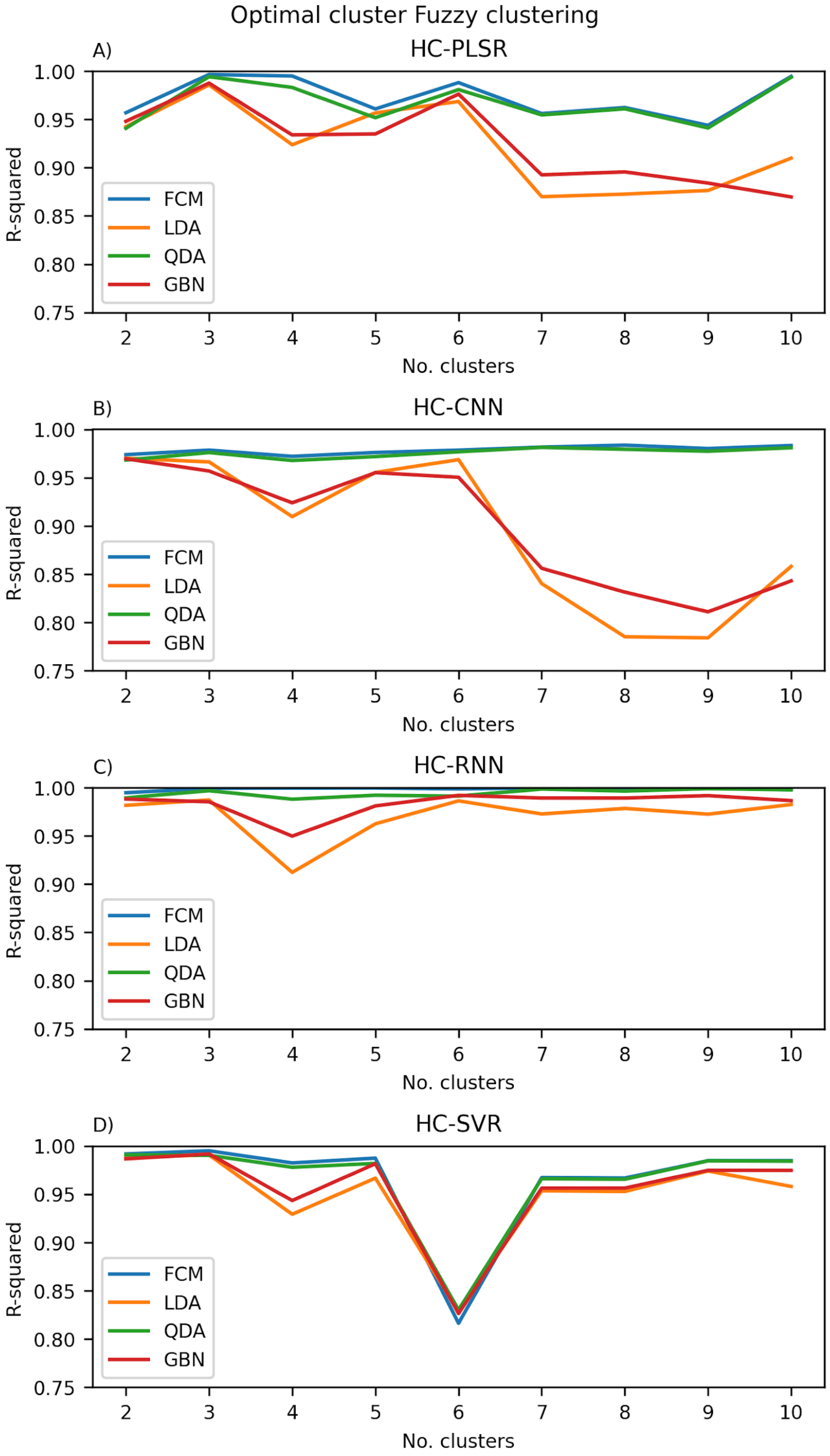

Figure S8 R^2 versus the number of clusters when using Fuzzy clustering for HC-PLSR (A), HC-CNN (B), HC-RNN (C) and HC-SVR (D).

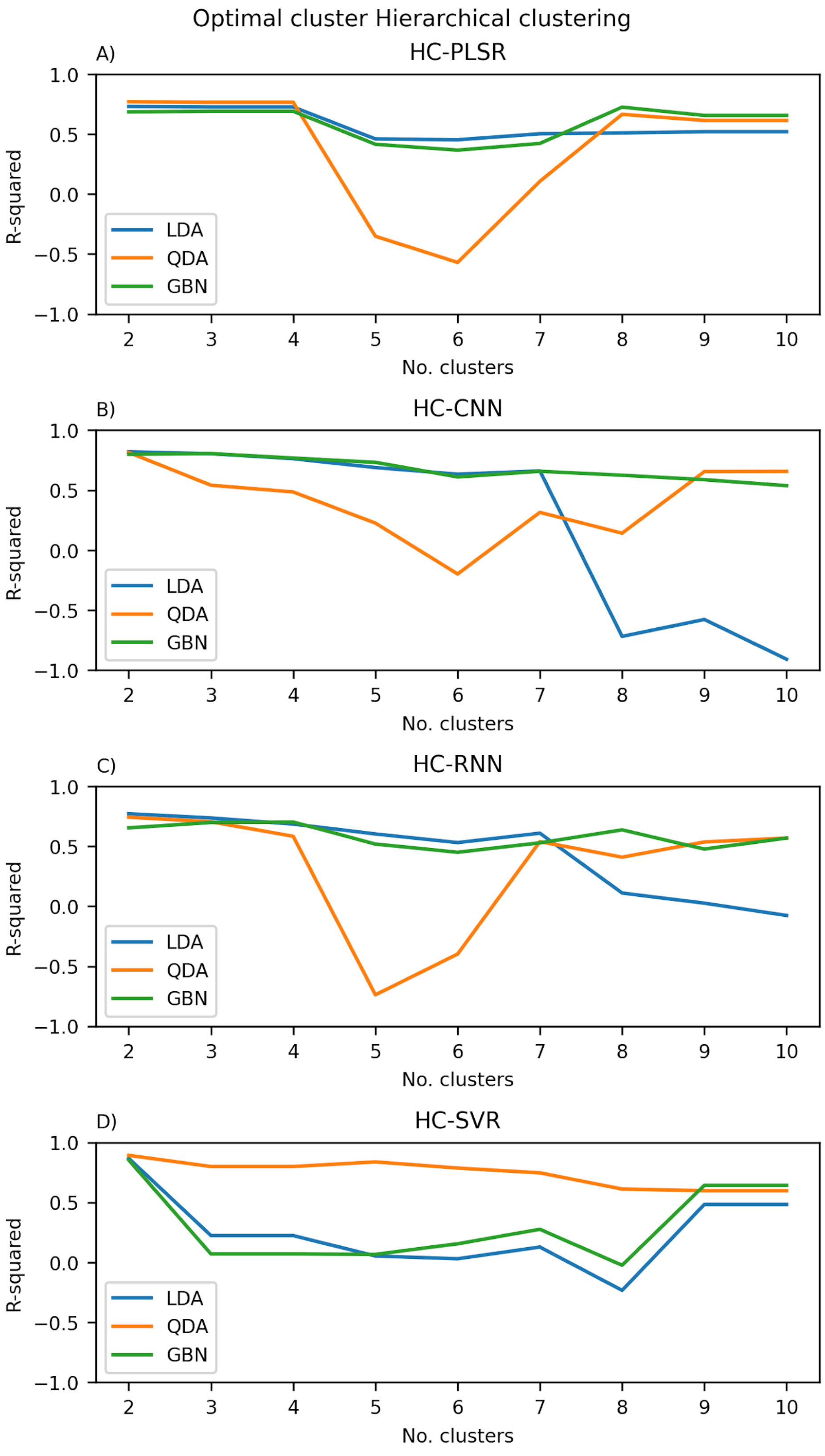

Figure S9 R^2 versus the number of clusters when using Hierarchical Agglomerative clustering for HC-PLSR (A), HC-CNN (B), HC-RNN (C) and HC-SVR (D).

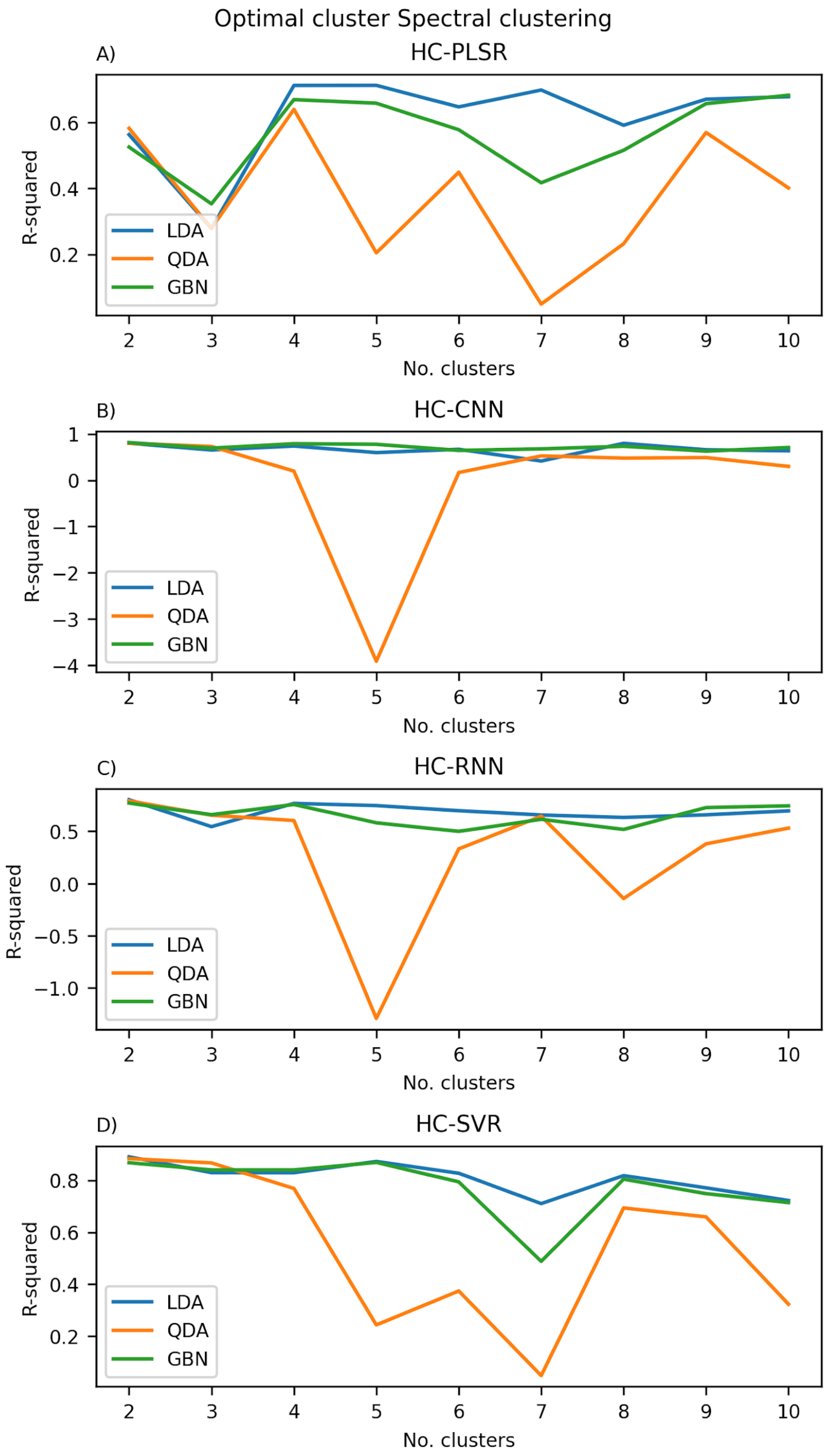

Figure S10 R^2 versus the number of clusters when using Spectral clustering for HC-PLSR (A), HC-CNN (B), HC-RNN (C) and HC-SVR (D).
